# Sex-Specific Impact of Ischemic Preconditioning on Tissue Oxygenation and Maximal Concentric Force

**DOI:** 10.3389/fphys.2016.00674

**Published:** 2017-01-05

**Authors:** Pénélope Paradis-Deschênes, Denis R. Joanisse, François Billaut

**Affiliations:** ^1^Département de kinésiologie, Université LavalQuébec, QC, Canada; ^2^Institut Universitaire de Cardiologie et de Pneumologie de QuébecQuébec, QC, Canada; ^3^Institut National du Sport du QuébecMontréal, QC, Canada

**Keywords:** blood flow restriction, muscle function, oxygenation, performance, sex differences, athletes

## Abstract

Prior peripheral hypoxia induced via remote ischemic preconditioning (IPC) can improve physical performance in male athletes through improved O_2_ delivery and utilization. Since females may have an innate protective mechanism against ischemia-reperfusion injury, and since muscle metabolism during contraction differs between sexes, it is relevant to examine the impact of sex in response to IPC to determine whether it is also ergogenic in females. In a randomized, crossover, single-blind study, we investigated muscle performance, hemodynamic and O_2_ uptake in strength-trained males (*n* = 9) and females (*n* = 8) performing five sets of 5 maximum voluntary knee extensions on an isokinetic dynamometer, preceded by either IPC (3 × 5-min ischemia/5-min reperfusion cycles at 200 mmHg) or SHAM (20 mmHg). Changes in deoxy-hemoglobin (Δ[HHb], expressed in percentage of arterial occlusion and considered an index of O_2_ extraction), and total hemoglobin (Δ[THb]) concentrations of the vastus lateralis muscle were continuously monitored by near-infrared spectroscopy. The metabolic efficiency of the contractions was calculated as the average force/Δ[HHb]_avg_ ratio. Cohen's effect sizes (ES) ± 90% confidence limits were used to estimate IPC-induced changes and sex differences. IPC increased total muscular force in males only (13.0%, ES 0.64, 0.37;0.90), and this change was greater than in females (10.4% difference, ES 0.40, 0.10;0.70). Percent force decrement was only attenuated in females (−19.8%, ES −0.38, −0.77;0.01), which was clearly different than males (sex difference: ES 0.45, −0.16;1.07). IPC also induced different changes between sexes for average muscle O_2_ uptake in set 2 (males: 6.4% vs. females: −16.7%, ES 0.21, −0.18;0.60), set 3 (males: 7.0% vs. females: −44.4%, ES 0.56, −0.17;1.29), set 4 (males: 9.1% vs. females: −40.2%, ES 0.51, −0.10;1.13), and set 5 (males: 10.2% vs. females: −40.4%, ES 0.52, −0.04;1.09). However, metabolic efficiency was not meaningfully different between conditions and sexes. IPC increased muscle blood volume (↑[THb]) at rest and during recovery between sets, to the same extent in both sexes. Despite a similar IPC-induced initial increase in O_2_ delivery in both sexes, males displayed greater peripheral O_2_ extraction and greater strength enhancement. This ergogenic effect appears to be mediated in part via an up regulated oxidative function in males. We conclude that strength-trained males might benefit more from IPC than their female counterparts during repeated, maximal efforts.

## Introduction

Ischemic preconditioning of a limb (IPC) is a non-invasive technique inducing transient peripheral hypoxia to subsequently enhance tissue tolerance against ischemia-reperfusion injury. This technique promotes local vasodilation, improves O_2_ delivery (Enko et al., 2011; Bailey et al., 2012a), and enhances the efficiency of muscular contraction (Pang et al., 1995; Moses et al., 2005). Higher muscle O_2_ utilization was also demonstrated in the quadriceps of strength-trained male athletes after performing IPC compared to placebo compressions (Paradis-Deschenes et al., 2016). It is not surprising, therefore, to observe that IPC can improve maximal physical performance in various exercise modes in male participants (De Groot et al., 2010; Bailey et al., 2012a; Paradis-Deschenes et al., 2016), although this is not a universal finding (Incognito et al., 2015). Before exercise sport performance applications, IPC was originally studied for its potential clinical relevance, and in this context, it is interesting to note that females appeared to display smaller clinical benefits compared with their male counterparts. In a cohort of 382 subjects, a failure to induce preconditioning effects during percutaneous coronary intervention was noted in females (Laskey and Beach, 2003), presumably due to the innate, multiple, protective actions of estrogens (Pitcher et al., 2005). Moreover, since physiological responses to exercise (in particular metabolic pathway utilization and perfusion) may differ between sexes, and that exercise performance data from females is almost inexistent, it is relevant and timely to examine the impact of IPC on females compared to males to more precisely evaluate the potency of this technique for exercise performance applications.

Females have been reported to exhibit less fatigue than males during intense exercise (Parker et al., 2007; Hunter, 2016), but not under conditions of ischemia (Russ and Kent-Braun, 2003), suggesting that muscle perfusion and oxygenation may be involved in the sex-related difference (Russ and Kent-Braun, 2003; Clark et al., 2005; Hunter et al., 2006). Indeed, females exhibit greater vasodilation in the limbs during single knee extensions (Parker et al., 2007), and may have a greater proportional area of type I fibers (Simoneau et al., 1985; Staron et al., 2000) and greater capacity for utilizing oxidative metabolism than males (Kent-Braun et al., 2002). Thus, females exhibit a greater reliance on oxidative metabolism compared with males, which could challenge the ergogenic impact of IPC.

Prior evidence examining sex-related differences in the response to IPC during exercise is limited. Gibson et al. (2015) reported no performance difference between male and female team-sport athletes during five repeated 6-s sprints after performing 3 × 5-min occlusions at 220 mmHg to both legs. However, the IPC procedure used in that study failed to improve performance in either sex, making the sex comparison moot. Another study demonstrated the positive impact of IPC (2 × 3-min at 220-mmHg) on recovery from squat jump test and running sprint performance 24 h after an initial, fatiguing session in males, but the female cohort was too small to draw any firm conclusion (Beaven et al., 2012). Considering the scarcity of studies and that none have attempted to measure relevant physiological responses to provide sex-specific mechanistic insights, any conclusion on the usefulness of IPC for female athletes cannot robustly be drawn.

The aim of the current investigation was therefore to determine the impact of IPC on muscle force and haemodynamics (blood volume and O_2_ extraction) derived from near-infrared spectroscopy in males vs. females during repeated maximal efforts separated with incomplete recovery periods. We chose isolated contractions specifically to avoid confounding effects that inspiratory muscle fatigue can have on limb blood flow and O_2_ uptake (Kayser et al., 1997).

## Materials and methods

### Participants

Strength-trained (power and weight lifters, cross-fit and taekwondo athletes) males (*n* = 9, age 25 ± 2 year; height 1.78 ± 0.02 m; weight 86.5 ± 4.9 kg) and females (*n* = 8, age 22 ± 1 year; height 1.66 ± 0.02 m; weight 60.8 ± 2.7 kg) volunteered to take part in this study. All performed 3–5 weight training sessions per week. All participants were non-smokers, free of health problems, did not use any medication, and were asked to avoid vigorous exercise, alcohol and caffeine 24 h before the tests. All but one female (who was amenorrheic) were tested in their follicular phase. Participants provided written informed consent after being informed of experimental procedures, associated risks and potential benefits. The study was approved by the Ethics committee of Université Laval, and adhered to the principles established in the Declaration of Helsinki.

### Experimental design

Participants visited the laboratory for one familiarization and two experimental trials. Resting heart rate and blood pressure (inclusion criteria <140/100 mmHg) were taken prior to every trial. During the first visit, height, weight and thigh circumference were measured. Thigh circumference (males: 61.3 ± 1.9 cm; females: 57.2 ± 1.9 cm) was measured by the same experimenter, 1 cm under the gluteal line. Participants then completed a familiarization session with the experimental set-up, comprising one 3-min compression at a pressure of 200 mmHg, and a standardized warm-up consisting of 5 min of cycling on a Monark ergometer (Ergomedic 828 E) at 100 W. The warm-up was continued with 3–5 right-leg extensions on an isokinetic dynamometer (Kin-Com 500 H, Chattecx Corp., Hixson, TN) at 20°/s, with effort perception progressing from 3 to 9 out of a scale of 10. After 2 min of rest while seated on the dynamometer, participants completed three complete sets of the exercise protocol described below.

Following familiarization, participants were randomized into IPC or SHAM groups in a single-blind, crossover design. In both conditions, participants were seated comfortably on a bed with both legs outstretched, and a non-elastic nylon blood pressure cuff (WelchAllyn, Skaneateles Falls, NY, USA, width: 21 cm) was positioned around the right upper thigh under the gluteal line. In IPC, the cuff was rapidly inflated to 200 mmHg for 5 min, and this was repeated three times with each compression episode separated by 5 min of reperfusion (cuff release) in the same position. A plateau in the NIRS-derived deoxy-hemoglobin concentration signal (see NIRS procedure below) was observed in every subject by 5 min, and taken as a sign of effective occlusion and ischemia. In SHAM, the cuff was inflated to 20 mmHg. To minimize any placebo effect, participants were told that the purpose of the study was to compare the impact of two different cuff pressures that could both alter performance.

The familiarization session and experimental trials were separated by a minimum of 3 days to eliminate the potential effects of the second window of protection caused by IPC (Bolli, 2000), and a maximum of 7 days. All trials were performed at the same time of day for every participant to avoid potentially confounding circadian rhythm effects. The laboratory temperature was controlled and constant (20.31 ± 0.02°C) throughout all trials.

### Exercise protocol

The exercise protocol started 18.5 ± 0.1 min after the end of the last cycle of compression. Participants were seated in an upright position on the isokinetic dynamometer, and a strap was secured tightly across the pelvis. The right leg was fixed to the dynamometer with a strap above the ankle external malleoli, and the axis of rotation was aligned to the lateral femoral condyle of the knee joint.

The protocol consisted of five sets of 5 maximum voluntary knee extensions (60° range of motion from 80 to 20°; 0° corresponding to knee fully extended) at 20°/s angular velocity (one extension lasting ~3.0 s). Participants were instructed to contract as hard as they could throughout the extension, and were strongly encouraged during all contractions. Contraction was stopped during flexion when the dynamometer arm automatically returned to 80° at angular velocity of 120°/s (lasting less than 0.5 s), and started immediately after the return of the arm. Subjects rested quietly and relaxed for 30 s between each set and after the last set. After the exercise, participants moved back to the bed to perform an arterial occlusion with the cuff at 200 mmHg (~3–5 min) to obtain a physiological calibration of the NIRS signals. The cuff pressure was released after the deoxy-hemoglobin signal had reached a plateau (see Near-infrared spectroscopy procedure below). Participants were also asked which condition between IPC and SHAM they felt had the greatest impact on their performance, and their verbal answer recorded.

The force produced by participants was measured with a force transducer connected at the end of the level arm of the dynamometer, which was calibrated according to the manufacturer's recommendations before every trial (manufacturer typical error 0.5%). The intra- and inter-day coefficient of variation for force obtained by the main experimenter was 2.4%. Force signals were analyzed in Matlab® between a starting point defined when velocity was ≥18°/s, angle was ≥80° and force was ≥100 N, and an end point when velocity was ≥18°/s and angle was ≥20°. In every set, peak and average force were calculated. Total force was then calculated as the sum of the average force produced in all sets. Percent force decrement across all sets was calculated as follows: 100 − ([total force output/ideal force output] × 100), where total and ideal force outputs are the sum of average force values from all sets and the highest average force was multiplied by five, respectively.

### Near-infrared spectroscopy (NIRS)

NIRS is a versatile, non-invasive methodology providing semi-quantitative measures of tissue oxygenation, and is easily applied to study a variety of tissue regions in individuals. NIRS quantifies the changes in hemodynamics from changes in the absorption of near-infrared light by oxyhemoglobin (HbO_2_) and deoxyhemoglobin (HHb) (Mccully and Hamaoka, 2000). With this technique, oxygenation can be measured in a discrete region of a tissue in a working physiological setting, which enhances specificity and has distinct advantages as compared to more cumbersome methods. Muscle tissue oxygenation measured by NIRS reflects the balance of O_2_ delivery to working muscles and muscle O_2_ consumption in capillary beds (De Blasi et al., 1993; Ferrari et al., 2004). Assessment of de- and re-oxygenation kinetics during and after dynamic exercise has become increasingly popular in recent years as a means to non-invasively assess the aerobic function of skeletal muscle. In the current protocol, muscle blood volume and oxygenation were assessed using a portable spatially resolved, dual wavelength NIRS apparatus (PortaMon, Artinis Medical Systems BV, Netherlands). The NIRS device was installed on the distal part of the right vastus lateralis belly (approximately 15 cm above the proximal border of the patella). Skinfold thickness was measured at the site of the application of the NIRS (males: 8.7 ± 0.9 mm; females: 10.7 ± 2.0 mm) using a Harpenden skinfold caliper (Harpenden Ltd) during the familiarization session, and was less than half the distance between the emitter and the detector (i.e., 20 mm). This thickness is adequate to let near-infrared light through muscle tissue (Mccully and Hamaoka, 2000). The skin was cleaned with an alcohol swab, and the device was fixed using double-sided stick disks and tape. Black bandages were used to cover the device to eliminate potentially interfering background light. The position of the apparatus was marked with an indelible pen for repositioning during the subsequent visit. The pressure cuff was positioned above the NIRS device, which did not affect the placement of the device during occlusions.

A modified form of the Beer-Lambert law, using two continuous wavelengths (760 and 850 nm) and a differential optical path length factor of 4.95, was used to calculate micromolar changes in tissue oxy-hemoglobin (Δ[HbO_2_]), deoxy-hemoglobin (Δ[HHb]) and total hemoglobin (Δ[THb] = [HbO_2_] + [HHb]; used as an index of change in regional blood volume). NIRS data were acquired at 10 Hz. At rest, once the signal was stabilized, 1 min of baseline values were analyzed pre IPC and SHAM treatments. Then, NIRS signals were analyzed 2-min post treatment for a duration of 1 min to assess the impact of IPC on resting blood volume (Δ[THb]_rest_, μM). During exercise, NIRS analysis was limited to Δ[HHb] since this variable is less sensitive than [HbO_2_] to perfusion variations and abrupt blood volume changes during contraction and recovery (De Blasi et al., 1993; Ferrari et al., 2004). The [HHb] signal was averaged over the last second of every contraction and over every set to obtain peak (Δ[HHb]_peak_, % arterial occlusion) and mean (Δ[HHb]_avg_, % arterial occlusion) O_2_ extraction, respectively. These [HHb] data were then normalized to express the magnitude of changes from baseline, and expressed in percentage of the maximal amplitude calculated during an arterial occlusion performed at the end of exercise. Contraction metabolic efficiency was calculated as the average force/Δ[HHb]_avg_ ratio. Finally, during recovery periods between exercise sets, the muscle reoxygenation rate (ΔReoxy, μM.s^−1^) was calculated as the rate of change in [HHb] from the end of the exercise set to the end of the subsequent recovery period (i.e., the recovery of [HHb]; Billaut and Buchheit, 2013). During this period, the amplitude of change in [THb] (Δ[THb]_rec_) was also analyzed.

### Statistical analysis

All data are reported as means ± standard error (SE) or percentage changes from SHAM. The IPC-SHAM differences within the same group and between sexes were analyzed using Cohen's effect sizes (ES) ± 90% confidence limits (Batterham and Hopkins, 2006; Hopkins et al., 2009). Except for Δ[THb]_rest_ and Δ[THb]_rec_, all variables were log-transformed prior to analysis (Hopkins et al., 2009). Magnitudes of difference between conditions were determined with an effect size of 0.2 set to evaluate the smallest worthwhile change. Standardized effects were classified as small (>0.2), moderate (>0.5) or large (>0.8). The effect was deemed “unclear” if chances of having better/greater or poorer/lower change in performance and physiological variables were both >5% (Batterham and Hopkins, 2006; Hopkins et al., 2009).

## Results

All 17 participants met all criteria, completed the entire protocol, and tolerated the IPC procedure without complications. None of the participants could tell what condition produced the greatest change in performance.

### Performance

**Figure 1:**
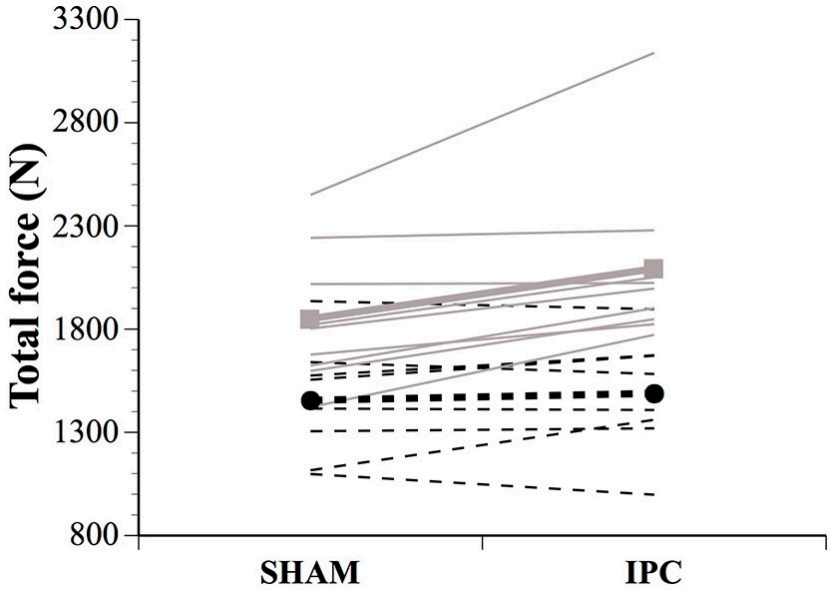
**Individual and average total force developed in SHAM and IPC conditions for males (■) and females (●)**. The IPC-induced change in total force was clearly higher in males than females. Values are mean ± SE.

**Figure 2:**
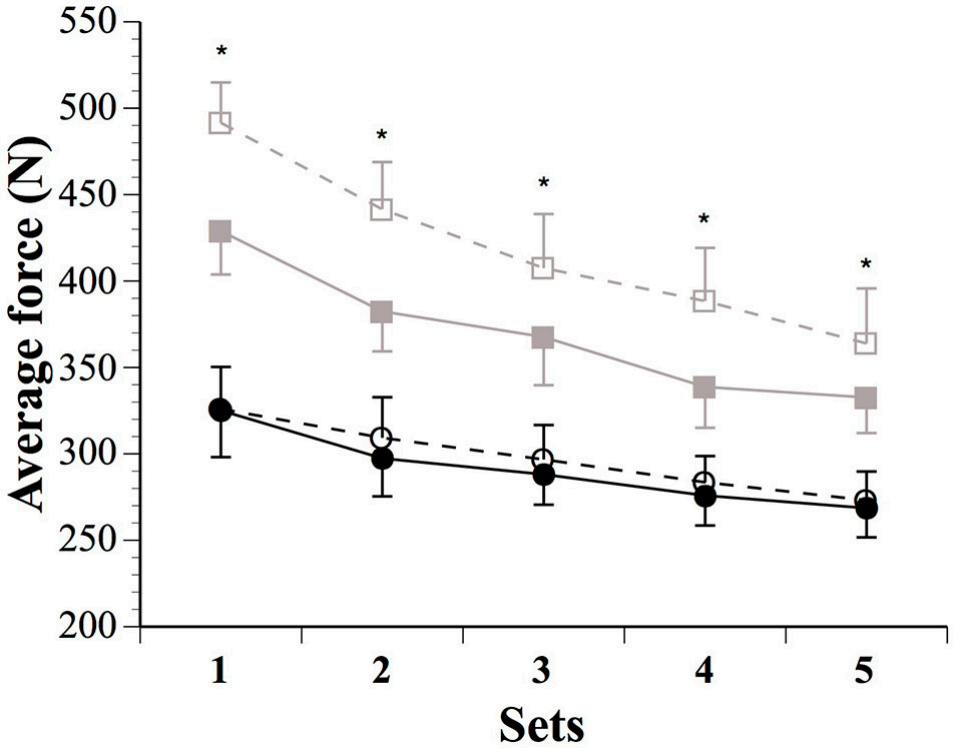
**Average force produced during the five sets in males (SHAM: ■ IPC: □) and females (SHAM: ● IPC: ○)**. Asterisks (^*^) denote “clear” differences between sexes (see statistics section). Values are mean ± SE.

Force parameters for males and females are displayed in Table 1 and Figures 1, 2. After the IPC manoeuver, total force clearly increased in males (13.0%, ES 0.64, 0.37;0.90), but the change was trivial in females (2.3%, ES 0.10, −0.17;0.38). &&&The sex difference for this parameter was clear (ES 0.40, 0.10;0.70; Figure 1). Specifically, the IPC-induced increase in average force was greater in males than females in every set of the protocol (set 1–males: 15.2% vs. females: 0.7%, ES 0.53, 0.16;0.90, set 2–males: 15.6% vs. females: 3.6%, ES 0.43, 0.10;0.76, set 3–males: 11.1% vs. females: 2.6%, ES 0.31, 0.02;0.61, set 4–males: 14.5% vs. females: 3.1%, ES 0.41, 0.07;0.76, set 5–males: 8.4% vs. females: 1.7%, ES 0.25, −0.06;0.56; Figure 2).

**Table 1:** **Performance variables in IPC and SHAM conditions for males and females**.

	**Females**			**Males**			**Sex difference**
	**SHAM**	**IPC**	**% difference (ES) 90% CL**	**SHAM**	**IPC**	**% difference (ES) 90% CL**	**(ES) 90% CL**
Peak force S1 (N)	449.1 ± 37.2	481.3 ± 42.1	7.2%, ES 0.24^*^, −0.07;0.56	627.9 ± 45.1	685.3 ± 35.7	10.2%, ES 0.41^*^, 0.23;0.59	ES 0.10, −0.24;0.44
Peak force S2 (N)	421.1 ± 32.8	446.3 ± 33.1	6.4%, ES 0.22^*^, 0.00;0.43	566.3 ± 31.3	648.9 ± 47.6	13.7%, ES 0.54^*^, 0.32;0.75	ES 0.24^*^, −0.03;0.50
Peak force S3 (N)	402.5 ± 22.7	429.5 ± 28.3	6.3%, ES 0.22^*^, 0.05;0.38	558.8 ± 35.7	581.6 ± 41.7	3.8%, ES 0.16, −0.01;0.32	ES −0.09, −0.29;0.12
Peak force S4 (N)	402.5 ± 24.7	422.0 ± 25.0	5.1%, ES 0.18, −0.06;0.41	526.3 ± 29.8	574.6 ± 43.2	8.4%, ES 0.34^*^, 0.06;0.61	ES 0.11, −0.20;0.42
Peak force S5 (N)	401.8 ± 27.1	397.5 ± 24.5	−0.7%, ES −0.02, −0.17;0.12	506.9 ± 28.3	544.0 ± 39.1	6.7%, ES 0.27^*^, 0.01;0.54	ES 0.26^*^, 0.00;0.51
Force decrement (%)	10.4 ± 1.5	8.4 ± 1.4	−19.8%, ES −0.38^*^, −0.77;0.01	13.7 ± 2.7	15.2 ± 2.2	6.0%, ES0.12, −0.54;0.77	ES 0.45^*^, −0.16;1.07

*Values are mean ± SE*.

Asterisks (^*^) denote “clear” effect sizes (see statistics section).

Importantly, although IPC did not increase average force in females, it clearly augmented 1-s peak force in sets 1–3 (Table 1), whereas males displayed benefits in sets 1, 2, 4, and 5. This effect was higher in males compared to females in sets 2 and 5.

Percent force decrement was attenuated in females after IPC (−19.8%, ES −0.38, −0.77;0.01), which was clearly different than males (sex difference: ES 0.45, −0.16;1.07).

### Muscle hemodynamics and oxygenation

Physiological variables are displayed in Table 2. No difference was observed between IPC and SHAM in both groups for NIRS variables during baseline before the manoeuver. IPC increased Δ[THb]_rest_ to the same extent in males and females (males: 1.5% vs. females: 1.5%, ES 0.00, −0.80;0.79). After IPC, Δ[THb]_rec_ was increased in males after sets 1 (1.2%, ES 0.33, −0.14;0.80) and 5 (1.5% ES 0.44, 0.17;0.70). In females, Δ[THb]_rec_ was only enhanced after sets 2 (0.7%, ES 0.23, −0.09;0.54) and 3 (1.8% ES 0.54, 0.04;1.04). This change in Δ[THb]_rec_ was higher in males compared to females in set 5 only (sex difference: ES 0.48, 0.10;0.87). There was no change in ΔReoxy within the same group and between sexes.

**Table 2:** **Muscle hemodynamic and oxygenation variables in IPC and SHAM conditions for males and females**.

	**Females**			**Males**			**Sex difference**
	**SHAM**	**IPC**	**% difference (ES) 90% CL**	**SHAM**	**IPC**	**% difference (ES) 90% CL**	**(ES) 90% CL**
[HHb]_base_ (μM)	25.3 ± 1.3	25.2 ± 1.1	−0.20%, ES −0.01, −0.35;0.33	37.5 ± 2.7	37.8 ± 2.8	0.85%, ES 0.03, −0.14;0.21	−5.39;7.92
[THb]_base_ (μM)	46.1 ± 3.5	43.8 ± 3.3	−4.80%, ES −0.21, −0.50;0.08	72.2 ± 5.3	72.6 ± 5.6	0.42%, ES 0.02, −0.12;0.15	ES 0.16, −0.06;0.38
Δ[THb]_rest_ (μM)	0.1 ± 0.4	1.6 ± 0.5	1.5%, ES 1.06^*^, 0.13;1.99	3.6 ± 0.6	5.1 ± 0.8	1.5%, ES 0.69^*^, −0.17;1.55	ES 0.00, −0.80;0.79
Δ[THb]_rec_ S1 (μM)	7.3 ± 1.0	7.8 ± 0.7	0.53%, ES 0.16, −0.27;0.59	9.1 ± 1.1	10.3 ± 1.3	1.16%, ES 0.33^*^, −0.14;0.80	ES 0.21, −0.46;0.88
Δ[THb]_rec_ S2 (μM)	5.0 ± 0.8	5.8 ± 0.5	0.75%, ES 0.23^*^, −0.09;0.54	6.4 ± 0.9	6.5 ± 1.5	0.14%, ES 0.04, −0.50;0.58	ES −0.20, −0.88;0.48
Δ[THb]_rec_ S3 (μM)	3.7 ± 0.8	5.5 ± 0.8	1.77%, ES 0.54^*^, 0.04;1.04	6.0 ± 1.0	6.5 ± 1.4	0.57%, ES 0.16, −0.21;0.53	ES −0.40, −1.04;0.25
Δ[THb]_rec_ S4 (μM)	4.7 ± 1.1	5.2 ± 0.7	0.58%, ES 0.18, −0.24;0.59	5.3 ± 1.1	5.9 ± 1.0	0.64%, ES 0.18, −0.26;0.62	ES 0.02, −0.62;0.66
Δ[THb]_rec_ S5 (μM)	5.5 ± 0.9	5.6 ± 0.77	0.08%, ES 0.02, −0.23;0.28	3.9 ± 0.9	5.4 ± 1.1	1.54%, ES 0.44^*^, 0.17;0.70	ES 0.48^*^, 0.10;0.87
ΔReoxy (μM.s^−1^)	0.1 ± 0.0	0.1 ± 0.02	−21.0%„ ES −0.32, −0.89;0.26	0.2 ± 0.1	0.2 ± 0.1	−4.0%„ ES −0.05, −0.38;0.028	ES 0.21, −0.29;0.70
Δ[HHb]_peak_ S1 (%AO)	59.0 ± 5.5	45.8 ± 13.3	−29.5%, ES −0.97, −2.86;0.92	69.8 ± 5.4	73.9 ± 3.9	7.3%, ES 0.26, −0.38;0.90	ES 1.35, −0.92;3.61
Δ[HHb]_peak_ S2 (%AO)	72.6 ± 7.3	57.3 ± 11.7	−10.3%, ES −0.30, −0.91;0.31	79.0 ± 5.6	83.9 ± 4.3	7.4%, ES 0.27, −0.35;0.89	ES 0.58, −0.24;1.40
Δ[HHb]_peak_ S3 (%AO)	75.0 ± 7.8	60.7 ± 11.8	−42.8%, ES −1.55, −4.11;1.01	80.4 ± 5.7	85.5 ± 3.9	7.7%, ES 0.28, −0.36;0.92	ES 2.04, −0.99;5.06
Δ[HHb]_peak_ S4 (%AO)	71.7 ± 7.3	61.4 ± 11.4	−29.1%, ES −0.95, −2.42;0.51	79.7 ± 6.8	86.3 ± 4.2	10.5%, ES 0.38, −0.24;0.99	ES 1.43, −0.32;3.17
Δ[HHb]_peak_ S5 (%AO)	68.4 ± 5.7	58.8 ± 11.5	−33.7%, ES −1.14, −2.64;0.36	82.7 ± 7.1	86.2 ± 4.2	6.4%, ES 0.23, −0.40;0.86	ES 1.52, −0.27;3.31
CMER S1	10.5 ± 1.4	5.6 ± 4.3	−4.4%, ES −0.12, −1.84;1.59	13.2 ± 2.5	11.8 ± 1.2	−2.4%, ES −0.04, −0.49;0.41	ES 0.05, −1.42;1.52
CMER S2	5.7 ± 1.3	7.2 ± 9.2	23.3%, ES 0.57, −0.57;1.72	5.6 ± 0.6	6.1 ± 0.8	8.7%, ES 0.14, −0.21;0.49	ES −0.29, −1.29;0.72
CMER S3	5.1 ± 1.1	9.9 ± 5.7	22.2%, ES 0.55, −0.46;1.56	5.1 ± 0.6	5.2 ± 0.6	3.8%, ES 0.06, −0.24;0.37	ES −0.37, −1.26;0.52
CMER S4	4.9 ± 1.0	17.3 ± 8.8	72.4%, ES 1.49, −0.42;3.41	4.7 ± 0.6	4.9 ± 0.5	5.0%, ES 0.08, −0.25;0.41	ES −1.13, −2.75;0.49
CMER S5	4.8 ± 0.9	19.3 ± 11.4	70.5%, ES 1.46, −0.30;3.23	4.6 ± 1.8	4.5 ± 0.5	−1.6%, ES −0.03, −0.36;0.30	ES −1.25, −2.75;0.25

*Values are mean ± SE*.

Asterisks (^*^) denote “clear” effect sizes (see statistics section). AO, arterial occlusion; CMER, contraction metabolic efficiency ratio; base, baseline before the IPC manoeuver; ΔReoxy, reoxygenation rate of the muscle; S, sets.

**Figure 3:**
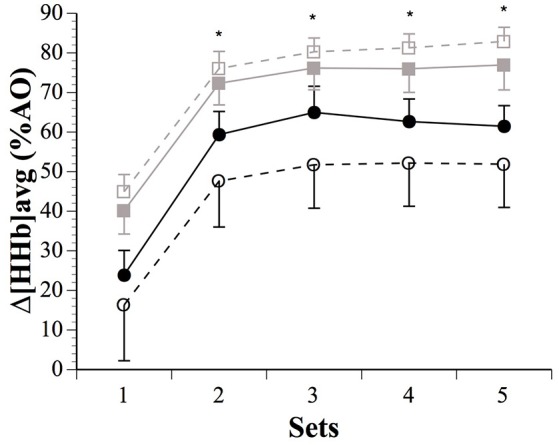
**Average changes in (Δ[HHb]_**avg**_), expressed as a fraction of the maximal values obtained during a transient arterial occlusion (AO) in males (SHAM: ■ IPC: □) and females (SHAM: ● IPC: ○)**. Asterisks (^*^) denote “clear” differences between sexes (see statistics section). Values are mean ± SE.

The IPC maneuver did not alter muscle Δ[HHb]_peak_ across sets nor between sexes (Table 2). However, Δ[HHb]_avg_ were higher after IPC in males for set 1 (18.1%, ES 0.31, −0.11;0.73), and lower in females for sets 3 (−44.4%, ES −0.25, −0.61;0.11), 4 (−40.2%, ES −0.22, −0.52;0.08), and 5 (−40.4%, ES −0.22, −0.50;0.06). There was a clear sex difference in the impact of IPC on global muscle deoxygenation in sets 2–5 (Figure 3). The contraction metabolic efficiency ratio was not altered by IPC during the sets or between sexes (Table 2).

## Discussion

### Summary of main findings

This study investigated the impact of sex on performance and vasoactive and oxidative responses to IPC in strength-trained athletes during repeated, maximal contractions. The main findings were that IPC (1) increased muscle force to a greater extent in males than females; (2) increased resting blood volume similarly in both sexes; and (3) increased O_2_ extraction in males but decreased it in females. These results challenge the general applicability of IPC on physical performance. While it may be recommended to enhance exercise capacity in males, this preconditioning technique appears less effective in females during maximal efforts.

### Muscle force parameters

IPC affected muscle force production and the ability to resist neuromuscular fatigue differently between sexes. While male athletes increased peak and average maximal concentric force in every set of the protocol following IPC, the benefits in females were lesser (Figures 1, 2). Females only displayed small improvements in peak force in the first three sets, while average force changes were trivial across all sets. Importantly, while IPC yielded acute positive responses in every male, four females out of eight experienced a decrease in performance. Another study did not report any sex difference in repeated-sprint performance, but the IPC protocol employed did not yield any positive effects in either males or females and, therefore, sex-related differences could not be truly assessed (Gibson et al., 2015). Although not studying sex differences *per se*, Gibson and colleagues (Gibson et al., 2013) reported altered 30-m running sprint times after IPC in females, while males displayed no added benefit. Taken together, these results highlight the importance of considering inter-individual responses to IPC in sports, particularly in female athletes. Considering the complex sex modulation of preconditioning mechanisms (such as mitochondrial K_ATP_ channel activation, reactive oxygen species generation, nitric oxide synthase activity, and inflammatory mediator production), as well as robust data documenting that females experience an innate protective mechanism after several forms of acute injury (for review see Pitcher et al., 2005), one could expect a sex-specific impact of remote IPC on physical performance. That said, both sexes are capable of being preconditioned (Pitcher et al., 2005). Hence, it is possible that females in the current study did not reach a sufficient threshold for preconditioning to occur. Thus, contrary to the proposition of a responder vs. non-responder pattern (Beaven et al., 2012; Gibson et al., 2013, 2015), the current data coupled with other clinical studies rather suggest that females may require a greater stimulus for effect. This remains to be elucidated by investigating the influence of varying numbers of IPC cycles and/or the number of limbs occluded at one time.

The sex-specific impact of IPC on fatigability has not been robustly assessed in the literature. The present data demonstrated clear differences between sexes; compared with males, females exhibited a lower force decrement over the five sets of maximal, isokinetic contractions. The lack of change in males is in keeping with previous studies reporting no differences between SHAM and IPC conditions in the measured fatigue index, despite higher peak and mean power outputs during the first repetitions of a series of ten 6 s cycle sprints (Patterson et al., 2015), or higher average force during maximal voluntary knee extensions after IPC (Paradis-Deschenes et al., 2016). This apparent sex difference in response to IPC could be attributed in part to the fact that males increased their initial and total force leading to greater subsequent metabolic and ionic perturbations (Balsom et al., 1994; Glaister, 2005). However, this cannot be the only explanation, as females clearly improved their resistance to fatigue across the sets by approximately 2%. Sex differences in the availability and use of O_2_ could shed some light on these differing responses to IPC.

### Muscle hemodynamic and oxygenation

IPC is known to increase blood flow in both ipsilateral (Kraemer et al., 2011) and contralateral limbs (Enko et al., 2011). It also up-regulates endothelial function at rest (Moro et al., 2011), after local transient ischemia (Kharbanda et al., 2001; Loukogeorgakis et al., 2005), and prevents the decline in flow-mediated dilation observed after strenuous exercise (Bailey et al., 2012a) in males. By investigating the NIRS-derived [THb] changes from pre- to post-IPC, we confirmed the acute increase in local blood volume at rest in males, and extended this hyperperfusion finding to females. In fact, these moderate to large hemodynamic changes from baseline were similar in both sexes, of a magnitude of 1.5%. Such data from female participants are very scarce in the literature, and results do vary. While Kharbanda and colleagues reported no sex difference in flow-mediated dilation response during ischaemia-reperfusion after IPC (Kharbanda et al., 2001), the same response was found to be higher in females compared with males immediately post-preconditioning (Moro et al., 2011). There is, however, stronger evidence of a sex difference in vasodilator responsiveness. Females display higher brachial artery flow-mediated dilation (Levenson et al., 2001) and forearm vasodilatory response to acetylcholine and β2-adrenergic receptor stimulation (Dietz, 1999; Kneale et al., 2000) relative to males. At first glance, these sex-based differences in intrinsic physiological responses could explain the differing impact of IPC on performance in males vs. females observed in the current study. Greater effects on endothelial function should improve contractile activity (and/or efficiency) by allowing a better O_2_ supply to skeletal muscles during intense exercise. It is not clear why males had a greater increase in muscle force than females for a similar percent increase in Δ[THb]_rest_, but one could argue that males might benefit more than females from an up-regulated endothelial function since they possess a lower intrinsic vasodilator responsiveness. Nevertheless, NIRS does not offer a robust assessment of blood flow since it does not detect change in blood velocity (Delorey et al., 2003). Studies using Doppler ultrasound are warranted to investigate vasodilation and potential blood flow changes following IPC in males and females.

An augmented blood volume before exercise could facilitate O_2_ delivery to active skeletal muscles. While IPC-induced changes in peak muscle O_2_ extraction were trivial in males, they displayed meaningful changes in Δ[HHb]_avg_ across the sets (Figure 3). Males extracted more O_2_ than females after IPC in sets 2–5, with clearly increased muscle force. IPC has been reported to increase systemic maximal O_2_ uptake (De Groot et al., 2010), as well as local tissue deoxygenation at task failure during handgrip exercise at 45% maximal voluntary contraction (Barbosa et al., 2015), and decrease blood lactate accumulation during submaximal running exercise (Bailey et al., 2012b). IPC also accelerates muscle deoxygenation dynamics and enhances performance during whole-body cycling and sustained isometric contraction of the knee in males (Kido et al., 2015; Tanaka et al., 2016). However, despite large Δ[THb]_rest_, IPC decreased Δ[HHb]_avg_ in females in the current study. The above studies exclusively recruited males, thus the current study adds to the literature by demonstrating that IPC might not induce similar metabolic responses in female athletes. Although we did not measure blood flow *per se*, the similar Δ[THb]_rest_ after IPC in both sexes suggest that the sex difference in O_2_ extraction might not be directly related to a difference in O_2_ availability. Furthermore, intramuscular pressure is positively correlated with contraction intensity, and it is accepted that occlusion of muscle blood flow occurs at 50–60% maximal voluntary contraction (Wigmore et al., 2004), thereby limiting its impact on sex differences observed in O_2_ metabolism when contractions are performed maximally as in the current study. In fact, the excessive intramuscular pressures of the contractions made our [THb] data collected during contractions unusable. And along this line of reasoning, it is of note that sex differences in fatigue development and performance disappear when blood flow is occluded (Maughan et al., 1986; Yoon et al., 2007). This could suggest that O_2_ availability does not explain the current finding of upregulated O_2_ extraction in males only. Caution is of course needed when interpreting NIRS-derived results due to methodological confounding factors such as subcutaneous fat layer thickness (although it was below the recommended emitter-receptor distance in both sexes) and possible NIRS sensor movement on the skin. Finally, muscle re-perfusion and thereby re-oxygenation occurring between maximal efforts is correlated with the recovery of muscle performance mainly via the resynthesis of phosphocreatine and by-products removal (Kime et al., 2003; Billaut and Buchheit, 2013). However, sex differences in Δ[THb]_rec_ were mostly unclear, as was the case for ΔReoxy. Based on these data, the sex-specific impact of IPC on exercise performance does not appear to be attributed to recovery processes.

There is also the possibility of a preferential impact of IPC on type II muscle fibers. A lower proportional area of type I fibers has been found in the vastus lateralis of males compared with females (Simoneau et al., 1985; Staron et al., 2000). Type II fibers display greater fractional O_2_ extraction with faster kinetics and lower microvascular O_2_ partial pressure (i.e., better muscle O_2_ diffusion index), despite a lower overall O_2_ consumption (Mcdonough et al., 2005). Interestingly, extraneous infusion of adenosine, which is a key acting molecule released during IPC, preferentially enhances vasodilation of arterioles to type II fibers (Wunsch et al., 2000). Therefore, due to the greater O_2_ extraction of type II fibers when highly perfused (Wilson et al., 1977), it has been suggested that these fibers might benefit more from an increase in blood perfusion than the more aerobic type I fibers (Faiss et al., 2013; Paradis-Deschenes et al., 2016), which could explain the higher Δ[HHb]_avg_ in strength-trained males in the present study.

In conclusion, this applied study demonstrated that strength-trained males might benefit more clearly from IPC than their female counterparts during repeated, maximal contractions. This strengthens clinical observations that sex may be a confounder in the response to this stimulus. Despite a similar increase in blood volume (↑[THb]) in both sexes immediately after IPC, and thus presumably similar increase in O_2_ availability, males displayed greater peripheral O_2_ extraction (↑Δ[HHb]). This ergogenic effect therefore appears to be mediated in part via an upregulation of oxidative function in males, possibly within type II muscle fibers.

## Author contributions

PPD, DRJ, and FB conceptualized and designed the research project; PPD acquired the data and conducted the statistical analysis; PPD interpreted results with assistance from DRJ and FB; PPD wrote the manuscript with revisions from DRJ and FB. All authors reviewed and agreed upon the final manuscript.

### Conflict of interest statement

The authors declare that the research was conducted in the absence of any commercial or financial relationships that could be construed as a potential conflict of interest.
